# Nanopasta: electrospinning nanofibers of white flour†

**DOI:** 10.1039/d4na00601a

**Published:** 2024-10-30

**Authors:** Beatrice Britton, Fangyuan Zhang, David B. Anthony, Ceasar III D. L. Reyes, Michal Pawlus, Gareth R. Williams, Adam J. Clancy

**Affiliations:** a Department of Chemistry, University College London London WC1E 6BT UK a.clancy@ucl.ac.uk; b UCL School of Pharmacy, University College London London WC1N 1AX UK g.williams@ucl.ac.uk; c Department of Chemistry, Imperial College London London W12 0BZ UK

## Abstract

White flour may be directly electrospun, providing a starch nanofiber alternative which avoids unnecessary industrial extraction and purification. By dissolving 17 wt% flour in warm formic acid and cooling, a dope can be created which can be electrospun into porous mats of 372 nm fibers of pasta.

## Abstract 2

We report ultrafine fibres prepared from wheat flour: a starch-rich nanoscale mesh with the potential in applications from biomaterials to filtration, without the extensive environmentally-damaging purification steps involved in starch production.

## Graphical abstract

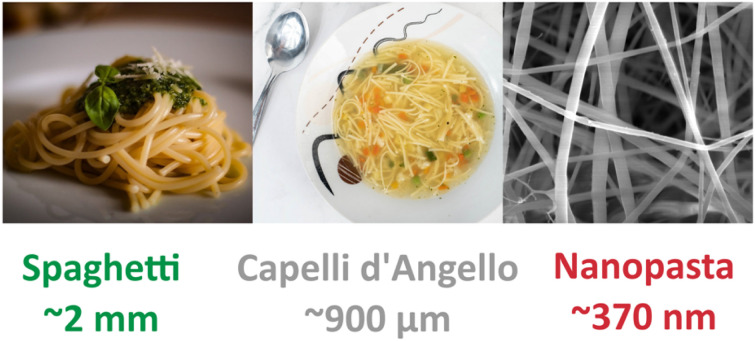

As the one of the most abundant natural polymers, starch has attracted interest across many applications, from biofuels^1^ to cosmetics^2^ to papermaking.^3^ Beyond these additive/precursor uses, the assembly of starch into bulk materials holds promise, notably as nanofiber membranes which may be used for nanofiltration,^4^ carbonized supercapacitor electrodes,^5^ or a host of biomedical applications. For the latter, the intrinsic high surface area and flexibility of nanofibers is combined with starch's biodegradable/biocompatible nature, flexibility of chemical modification, and reasonable mechanical properties to provide a platform for drug delivery,^6^ bone regeneration scaffolds,^7^ and wound healing.^8^

Fibers of starch may be created through a number of classic fiber assembly processing routes including extrusion^9^ and wet-spinning^10^ to give microscale fibers. However, these fibers have lower specific surface area and per-fiber tensile strength than starch nanofibers (*i.e.* with diameter < 1 μm), which are near-universally assembled through electrospinning: applying an electric charge to a starch solution which is ejected towards a grounded substrate while drying during flight, forming a mat of deposited starch fibers. A suitable dope for electrospinning is determined by a variety of factors including conductivity, volatility, surface tension, homogeneity, viscosity of the initial solution, and, relatedly, sufficient polymer entanglements to maintain a cohesive fiber structure during the spinning. The creation of the precursor starch dope is complicated by the intrinsic chemistry of starch,^11^ which consists of α-d-glucose linked *via* α(1 → 4) glycosidic bonds to form either linear chains of several hundred repeat units, termed amylose (∼20 wt%), or a branched structure with chains linked by additional regular α(1 → 6) glycosidic bonds, termed amylopectin (∼80 wt%), consisting of several thousand glucose units. The abundance of hydroxyls in starch leads to each molecule adopting a helical configuration held together by hydrogen bonding, with amylose forming rods and amylopectin arranging into lamella of locally parallel double-helices, separated by amorphous regions. Together, the components assemble into 1–100 μm granules with concentric semi-crystalline and amorphous regions. These coils and granules must be disrupted to enable the polymer entanglement necessary for electrospinning.

Few solvent systems are known to be suitable for disrupting the starch structure to create a spinnable dope, including dimethyl sulfoxide,^12^ aqueous sodium hydroxide, and ionic liquids.^13^ One solvent of note is formic acid (FA) which has complex temporal behavior, as described by Lancuški *et al.*^14^ Here, the addition of FA to starch initially begins uncoiling the starch and breaking apart the macroscopic starch granules, primarily through formylation of the hydroxyls to formate esters but also concurrent glycosidic bond cleavage. After several hours at room temperature, sufficiently high concentration solutions will gel from starch uncoiling sufficiently for entanglement, but will subsequently precipitate with further ageing, with fully formylated starch reforming a coiled structure. The rate of formylation (and hydrolytic depolymerization) are highly temperature, time, and FA concentration dependent,^15^ and varies between polymer types in the heterogeneous mixtures.^16^

Commercial starch production involves additional industrial steps to separate the non-starch components from a plant-derived source, such as steeping in SO_2_ solution to disrupt protein matrices surrounding starch, liberal washing, separation to remove solubilized contaminants, and drying the remaining starch solids. These processes^17^ require significant energy (160 kW h ton^−1^) and water (10 000 dm^3^ ton^−1^) which is contaminated with HCl, Na_2_SO_4_, and KOH during processing, in addition to high infrastructural cost. These starch extraction steps are undertaken to remove non-starch components such as proteins and cellulose from starch-rich plant matter. However, the removed components are biocompatible and biodegradable, and while the use of purified starch provides a comparatively simple model system, the removed impurities are often not intrinsically detrimental to the applications of starch(-based) nanofibers. As such, a potential alternative to pure starch (nano)fibers is to use a starch-rich precursor without purification and its associated environmental cost.

One of the most common starch-rich plant matter is wheat flour, produced by grinding wheat seeds to give whole-grain flour consisting of a fine powder of ground endosperm and more coarsely ground germ and bran. The endosperm component may be separated by sieving to give “refined” or “white” flour. The flour itself is a complex heterogeneous mixture of compounds which vary between the specific plant sources. As an example, durum wheat consists of ∼80% starch, ∼15% protein, ∼4% non-starch polysaccharides, and ∼1% fats. The proteins are predominantly glutens, which are a complex family of proteins, broadly split into glutenins (proteins networked by disulfide bridges) and gliadins (single molecule proteins). The constituent proteins have a large range of chemistries and molecular weights,^18^ with larger glutenin aggregates being several million Da, and gliadins around 28–55 kDa. The total energy cost of flour production is less than starch extraction^19^ (∼60 kW h per tonne, including preparation, milling, packaging, and transport) and does not contaminate water. The process is also routinely undertaken at large scale using established infrastructure.

Cylindrical fibers of wheat are well established culturally and industrially, known as the *pasta lunga* subcategory of pasta.^20^ While pasta may be made from combining flour with egg (*pasta fresca*), most commonly it is produced as *pasta secca* (dry pasta), extruded from a water-flour mixture and dried under controlled atmosphere to a desired internal water content, typically 12%, for long term storage. These dried fibers are later refluxed in ∼0.1–0.3 M sodium chloride aqueous solution, leading to swelling of amylopectin lamellae and subsequent unwinding of the amylopectin helices upon hydration, facilitating digestion. The nomenclature varies with the diameter of the fibers (and region), including ∼2 mm *spaghetti* (small string), ∼1.75 mm *vermicellini* (little worms), and ∼900 μm *capellini* (little hairs). The narrowest diameter mass-produced pasta is ∼800 μm *capelli d'angello* (angel hair), although thinner *pasta lunga* is produced by hand exclusively in the town of Nuoro, Sardinia: *su filindeu* (threads of God), which is estimated to have half the diameter of *capelli d'angello* and is, to the authors' knowledge, the thinnest pasta created by hand to date (∼400 nm thick pasta films have been previous created *via* glancing angle deposition of flour).^21^

Here, we use white flour as a nanofiber precursor, avoiding the often-unnecessary starch purification processing steps (Fig. 1). To the authors knowledge, the electrospinning of flour has not been performed previously: creating a suitable spinning dope from flour is a more challenging prospect than pure starch, owing to the more heterogeneous composition – notably the addition of gluten proteins and presence of cell walls which contain (hemi-)cellulose. Exploratory trials were attempted with established starch solvents to create electrospinning flour dopes, and only FA showed promise. Preliminary electrospinning parameters were determined using maize starch in FA, which was used as a baseline for comparison to flour nanofibers. Initial addition of FA to starch at room temperature formed a translucent gel with starch grains seen under optical microscopy, which persisted for 6 h. After this point, the viscosity decreased and a homogeneous transparent solution was seen, until ∼12 h after initial dissolution, whereupon a white precipitate formed. Electrospinning of solutions in the 6–12 h ageing window was performed, and continuous high quality nanofiber mats could be formed from dopes between 16–18 wt% in our setup, with an optimal concentration of 17 wt% (ESI, Note 1†).

**Fig. 1:**
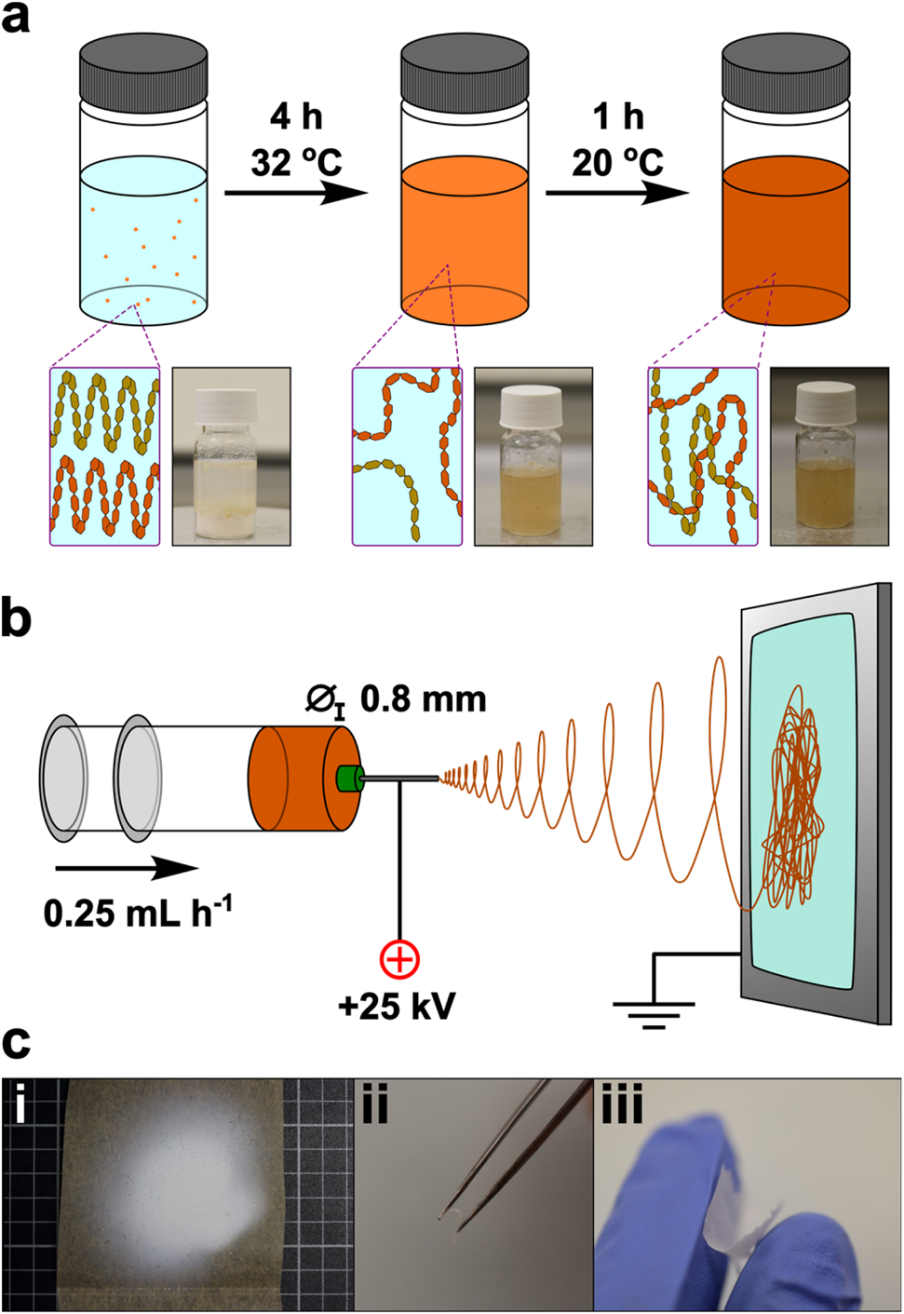
(a) Schematic of flour dope synthesis with diagrammatic breaking up of initial starch structure to free chains, with subsequent entangling on cooling, accompanied by digital photographs. (b) Schematic of electrospinning process. (c) Digital photos of (i) electrospun nanofiber mat, (ii and iii) freestanding materials bent to highlight flexibility and freestanding nature.

However, the use of flour at these weight fractions in FA led to a dope excessively viscous for electrospinning after dissolution at room temperature, *e.g.*, having a zero-shear-rate viscosity of 28.7 Pa s after 6 h, *versus* 10.3 Pa s for pure starch. The excessive viscosity was present regardless of ageing time and is attributed to the contribution from gluten which is known to directly be soluble in FA.^22^ Reduction of the flour loading formed solutions within the requisite viscosity (12 wt% – 8.9 Pa s; 13 wt% – 9.8 Pa s; 14 wt% – 11.1 Pa s) but led to electrospraying and dripping due to insufficient polymer entanglement.^23^

Instead, 17 wt% flour samples were dissolved at 32 °C to reduce viscosity while remaining below the 40 °C threshold known to induce significant hydrolysis.^16^ Under these conditions, initial addition of FA to flour led to formation of macroscopic aggregates which dissipated after ∼3 h, leaving a clear brown dope which was retained for 7 h, before onset of precipitation. The warmed solution 4 h after dissolution was insufficiently viscous for electrospinning (5.7 Pa s) but cooling to room temperature over 1 h gave a spinnable dope (11.0 Pa s). The mats were formed by electrospinning for 30 min, and formed a cohesive off-white film which could be removed as a free-standing sheet, although some samples showed mud-cracking after electrospinning indicative of drying of the sample on the surface. The mat consisted of fibers typically ranging from ∼100 to 600 nm in diameter, with an average of 372 ± 138 nm (Fig. 2f). The nanofiber surfaces were smooth (Fig. 2a–e) and continuous, suggestive of regular polymer arrangement(s), analogous to pure starch/FA-derived nanofiber mats (ESI, Fig. S6†). This change is ascribed to the facile rearrangement of polymers with interpolymer polar interactions, aided by the lessened hydrogen bonding from formylation.

**Fig. 2:**
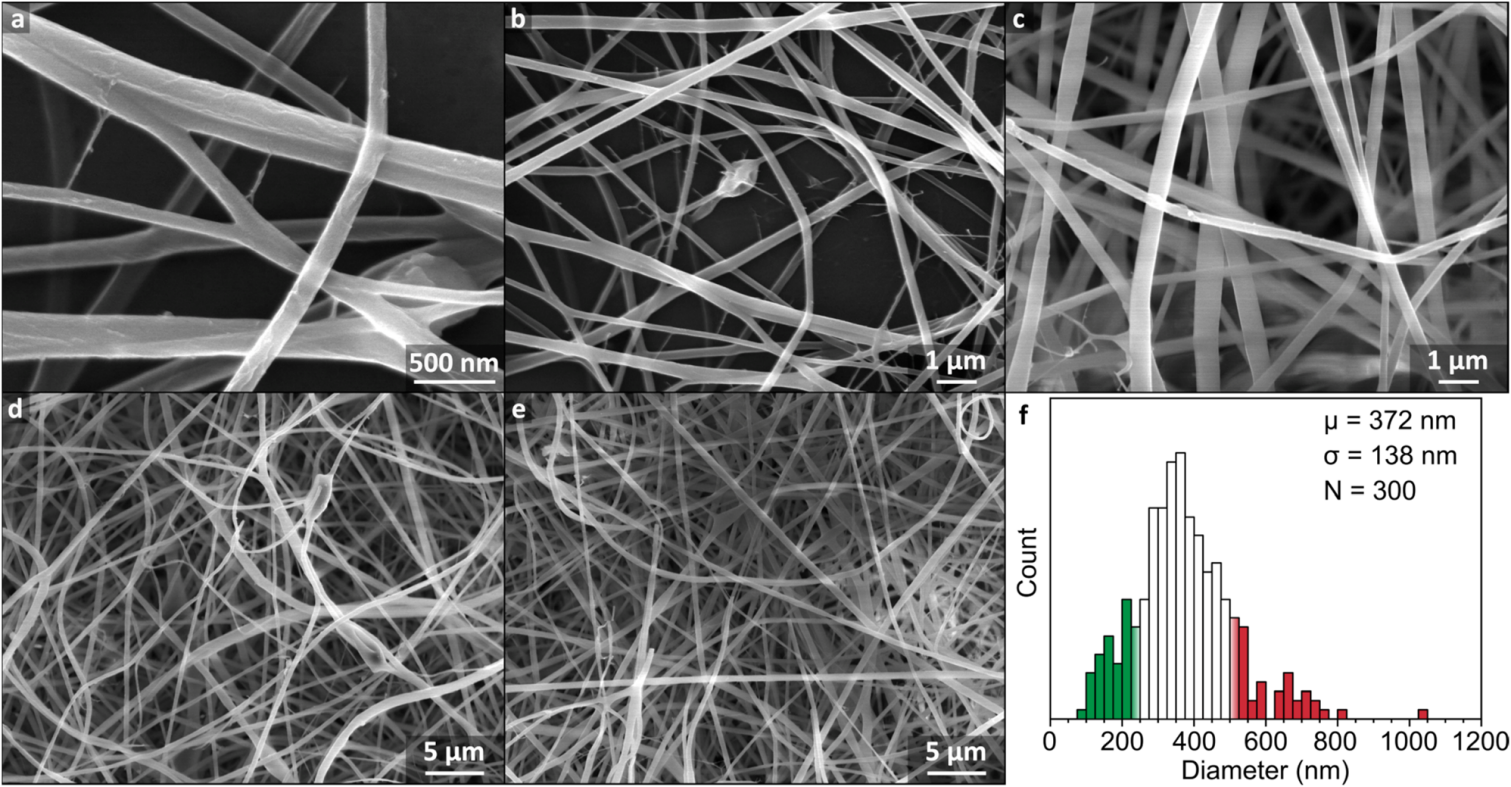
(a–e) SEM micrographs of nanofiber mat electrospun from 17 wt% flour dissolved in FA at 32 °C for 6 h and cooled for 1 h. (f) Histogram of fiber diameters with 25 nm bins with ±1 standard deviation from the mean given as coloured bars. Measurements are given in ESI Fig. S3.†

Formylation of hydroxyls was confirmed through a significant increase in the ester region carbonyl stretching mode (1710 cm^−1^) in the IR spectrum *versus* the initial flour (Fig. 3a), in addition to increases in the relative intensities of ethers (∼1150 cm^−1^) and C–O (1050 cm^−1^) stretches.^24^ The O–H hydrogen bonding peak area (∼3400 cm^−1^) of formylated flour is lower than the initial flour. While this smaller OH bonding IR peak may be in part attributable to formylation reducing the fraction of hydroxyls, it may also be linked to water loss as measured by thermogravimetric analysis (TGA, Fig. 3b) which shows a 6.5 wt% mass loss between 60–130 °C in the initial flour attributed to residual water, which is negligible for the electrospun flour (1.3 wt%). Importantly, this weight loss region also coincides with the boiling point of formic acid (101 °C), highlighting that excess formic acid is successfully removed from the material during the electrospinning procedure. Additionally, the mats lack FA's characteristic pungent odour (0.52 ppm detection threshold^25^), strongly implying that the small weight loss measured is adsorbed water. Starch degradation occurs between ∼250–350 °C and is the primary degradation in all cases. After formylation and electrospinning, the TGA of flour nanofibers directly demonstrates the formylation with an 18.6 wt% weight loss between 190–250 °C not seen in the initial flour, while the starch backbone weight loss remains near constant (54.0 wt% *vs.* initial 57.6 wt%). Assuming degradation through cleavage of HCOO, and the starch component is fully represented in the 250–350 °C loss (ESI, Fig. S2†), this corresponds to formylation of ∼1.1 hydroxyls per glucose unit. Unlike starch nanofibers which almost completely degrade to gaseous products by 600 °C (5.0 wt% remaining) in N_2_, there is a higher ash content in the flour nanofibers (13.3 wt% at 600 °C) attributed to the presence of oxygen-poor proteins. Starch packing can also be seen to have an impact as tightly-packed commercial starch has a higher ash content (14.8%, ESI Fig. S2a†) than the nanofibers, while initial flour consolidates both effects to give the highest ash content (23.3 wt%).

**Fig. 3:**
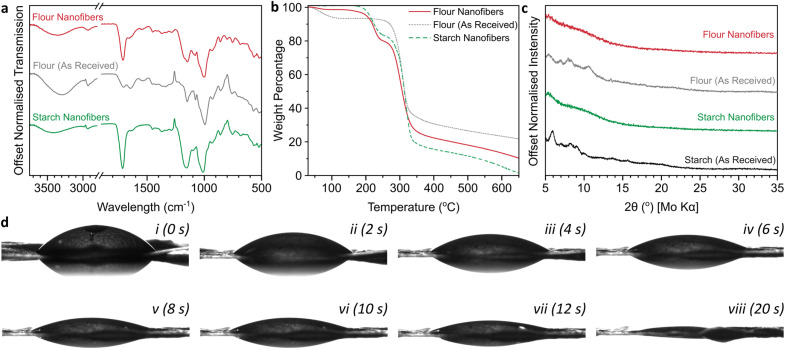
(a) ATR-IR spectra, (b) TGA thermograms under flowing N_2_ with 10 °C min^−1^ heating, derivatives provided in ESI Fig. S2,† (c) XRD diffractograms using Mo Kα (0.71 Å), (d) contact angle measurements of nanofiber mat (transferred to a glass slide) as a function of time in 2 s increments for the first 12 s (i–vii) and after 20 s (viii).

The fibers lose the limited crystallinity seen in the initial flour, as is also seen for starch samples (Fig. 3c) due to the degradation of the initial starch mesostructured during formylation. The films were hydrophilic, with sessile drop tests giving an initial contact angle of 53°, with the water absorbed into the film over several seconds (Fig. 3d, ESI Video S1†). The initial wettability is comparable to previous measurements of wheat flour,^26^ with the formylation not having a significant impact despite the reduction in fraction of hydroxyl groups. The absorption of water is attributed to the porous nature intrinsic to nanofiber mats and indicates that the flour fibers are a viable material for biological applications such as drug delivery and tissue engineering.

In contrast to the 17 wt% flour/FA solution, a reduction to 16 wt% led to insufficient polymer entanglement, giving dripping at low applied potential differences from high dope surface tension, and electrospraying at higher voltages from insufficient inter-polymer interactions to maintain jet shape (Fig. S4†). Conversely, 18 wt% flour/FA solutions were capable of forming nanofiber mats, albeit requiring higher voltages (22 kV), but fiber quality was low, with beading and droplets attributed to jet instability from a greater variation in local viscosity with a heterogeneous flour distribution (ESI, Fig. S5†).

Despite the general success of the electrospinning process, several local defects were seen throughout the sample (Fig. 2a–e). In a few local regions, droplets were present, indicative of temporary jet instability, while small quantities of nanonets were seen from the high necessary electrostatic repulsive forces causing branching jets.^27^ The flour fibers were seen to have both higher general curvature than the starch nanofibers (ESI, Fig. S6†), as well as distinct local buckles in some fibers, highlighting a greater instability of the spinning process for flour compared to starch. We attribute this difference to a greater inconsistency in dope viscosity of flour compared with the more homogeneous starch dopes, caused by a variation in size and composition of flour granules throughout the dope. We believe larger particles may have also contributed to the lower yield seen, with notable deposition of material seen on the container walls of the electrospinning set-up, consistent with clogging of the needle by larger residual flour particles.

## Conclusions

In conclusion, the electrospinning of wheat flour is possible from formic acid solutions, after ageing at 32 °C and cooling, forming mats of nanofibers with diameters of 372 (±138) nm. The formed mats are hydrophilic, and ideally positioned as a cheaper, greener replacement for starch in biodegradable, biosourced nanofiber applications, such as next generation bandaging, or carbonized supercapacitor electrodes. Additionally, as the newly developed material consists of fibers formed from the extrusion and drying of flour, it may be defined as pasta, dramatically undercutting the previous record for the thinnest *pasta lunga* by approximately a thousand times.

## Data availability

The data supporting this article have been included as part of the ESI.†

## Author contributions

Conceptualization – GRW, AJC; formal analysis – BB, DBA, AJC; investigation – BB, DBA, C3DLR, MP; methodology – BB, FZ; supervision – GRW, AJC; writing (original draft) – BB, AJC; writing (review & editing) – FZ, GRW.

## Conflicts of interest

There are no conflicts to declare.

## Supplementary Material

NA-OLF-D4NA00601A-s001

NA-OLF-D4NA00601A-s002

NA-OLF-D4NA00601A-s003
