# Supplementary material for: Nanopasta: electrospinning nanofibers of white flour
Source: Nanoscale Adv. 2024 Oct 30;6(24):6129–33. doi: 10.1039/d4na00601a (PMC11580803; doi:10.1039/d4na00601a)
Supplement: NA-006-D4NA00601A-s001 [file NA-006-D4NA00601A-s001.pdf]

Supplementary Information (SI) for Nanoscale Advances.
This journal is © The Royal Society of Chemistry 2024

Supplementary Information

Nanopasta: electrospinning nanofibers of white flour

Beatrice Britton, Fangyuan Zhang, David B. Anthony, Ceasar III D. L. Reyes, Michal Pawlus, Gareth R. Williams, Adam J. Clancy

Methods

Materials: Maize starch (73% amylopectin, 27 % amylose) and formic acid (≥95%) were purchased from Sigma-Aldrich (UK). Flour (Everyday Flour, Plain White Flour; carbohydrate 72.8 wt%, protein 9.9 wt%, fiber 2.6 wt%, fat 0.7 wt%, water 14.7 wt%) was purchased from the supermarket Marks and Spencer (UK).

Dope Synthesis: Dopes for electrospinning were created by taking a glass vial filled with a given amount of flour and under constant stirring with a PFTE magnetic stirrer (500 rpm), 10 mL of formic acid was added in 2 mL increments every 2 min before being left to stir at 21 or 32 °C (with the latter controlled by oil bath) for a given amount of time, with visual inspection using optical microscopy (EVOS FL, Life Technologies) taken on an aliquot every 1 h. For the 32 °C, the mixture was allowed to cool to room temperature over 1 h before use.

Electrospinning: A bench-top electrospinning set-up contained within a Pyrex enclosure contained a plastic syringe fitted with a 38.1 mm 21G needle (Nordson EFD, UK), connected to a syringe pump (KDS100, Cole Parmer) and a positive electrode (0-25 kV). Power was supplied using a FuG HCP20-20,000 generator (Germany). The collector plate, a grounded metallic plate wrapped in one sheet of baking paper, was positioned beneath the dispensing needle. Optimization of the electrospinning set-up for each solution was carried out by increasing the voltage from 0 – 25 kV in ~1 kV increments for a given flow rate (0.01 – 0.8 mL h^{-1}) and collector distance (5 – 12 cm), using a glass microscope slide for collection for 30 s for visual inspection of fibers under an optical microscope (EVOS FL, Life Technologies, 40 x magnification). Unsuccessful electrospinning was indicated by the collection of dripping on optical microscopy and successful spinning was indicated by the collection of fibers (Fig. S1). Optimized parameters for each sample are given in the Supplementary Table S1.

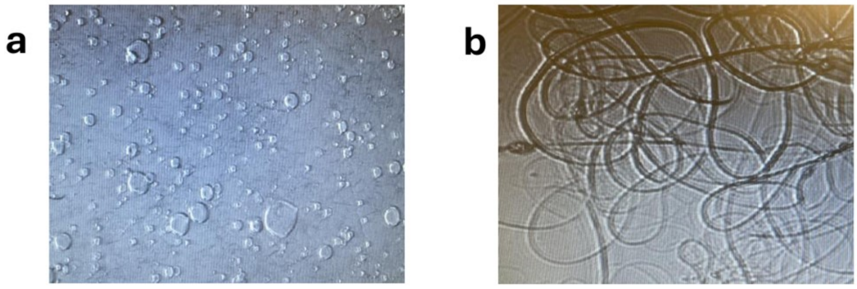

Figure S1. Representative examples of optical microscopy used to identify electrospinning parameter success, (a) droplets indicating unsuccessful parameters, (b) successful nanofiber formation.

Scanning Electron Microscopy: SEM was undertaken using a JEOL JSM-6701F scanning electron microscope (SEM) with an acceleration voltage of 10 kV. Samples were cut from the formed film with a fresh razor and adhered to an aluminium substrate with carbon tape and gold coated by sputter-coating for 10 s at 20 mA using an Agar Sputter Coater 108 prior to imaging.

ATR-IR: Spectra were collected on a Bruker Compact FT-IR Spectrometer Diamond ATR at room temperature between 340 cm^{-1} and 4000 cm^{-1} with 0.125 cm^{-1} resolution.

Thermogravimetric Analysis: TGA was performed on a PerkinElmer STA6000 TGA. Samples were placed in tared 150 µL alumina pans and held at 30 °C for 10 min under a 60 mL min^{-1} gas flow of nitrogen to purge air, before heating to 600 °C at a heating rate of 10 °C min^{-1}. Weights were normalized with the weight after the purge set to 100 wt%.

Goniometry: Nanofiber mat wettability was measured by a Kruss DSA25E Drop Shape Analyser, dispensing 3 µL of DI water onto flattened samples physically transferred to a glass slide. Initial contact angles are provided in Table S3, however, values decreased quickly with time in all samples as the samples absorbed water, and angles should not be taken as true values for hydrophobicity quantification.

Viscosity: Rheological measurements were undertaken with a Bohlin Gemini II Rheometer under steady-state shear flow. A parallel, 40 mm, plate was used with a gap of 0.6 mm and zero-shear viscosity was obtained from apparent viscosity against shear rate.

pXRD: X-ray diffraction measurements were undertaken on a Stoe STADI-P in thin foil mode using Mo Kα1 radiation (0.70930 Å, 50kV and 25mA). Samples were mounted between two amorphous acetate films, held with a small quantity of vacuum grease. Measurements were recorded between 2 – 40° 2θ in 0.5° steps for 15 s per step, while rotating the sample normal to the beam at ~60 rpm.

Tensile Testing: Tensile tests were carried out on a MFS350 Tensile Stress Tester (LINK, V2.1, Linkam Scientific Instruments Ltd., UK) in standard ambient atmospheric temperature and pressure using a 20 N or 2 N load cell at crosshead speed of 16 µm s^{-1}.

Table S1. Electrospinning parameters for each dope which led to successful nanofiber formation.

Dope	Collector distance (cm)	Flowrate (mL h^{-1})	Voltage (kV)
17 wt% Starch/FA	7	0.80	18 – 23
17 wt% Flour/FA	8	0.25	19 – 21
16 wt% Starch/FA	7	0.80	17 – 19
18 wt% Starch/FA	7	0.80	22 – 23
18 wt% Flour/FA	8	0.30	16.5

Table S2. Viscosity values for dopes of different nanofiber dopes of differing synthesis conditions.

Dope	Loading (wt%)	Synthesis Conditions	Viscosity (Pa s)
Stach	17	RT, 6 h	10.3
Flour	12	RT, 6 h	8.9
Flour	13	RT, 6 h	9.8
Flour	14	RT, 6 h	11.1
Flour	17	RT, 6 h	28.7
Flour	17	40 °C 4 h	5.7
Flour	17	(i) 40 °C 4h (ii) RT, 1 h	11.0

Table S3. Initial contact angles after initial droplet addition.

Sample	Initial Contact Angle (degrees)
Greaseproof paper (substrate)	112.4
Starch Nanofibers (17%/FA)	52.5
Flour Nanofibers (17%/FA)	46.0

Supplementary Figures

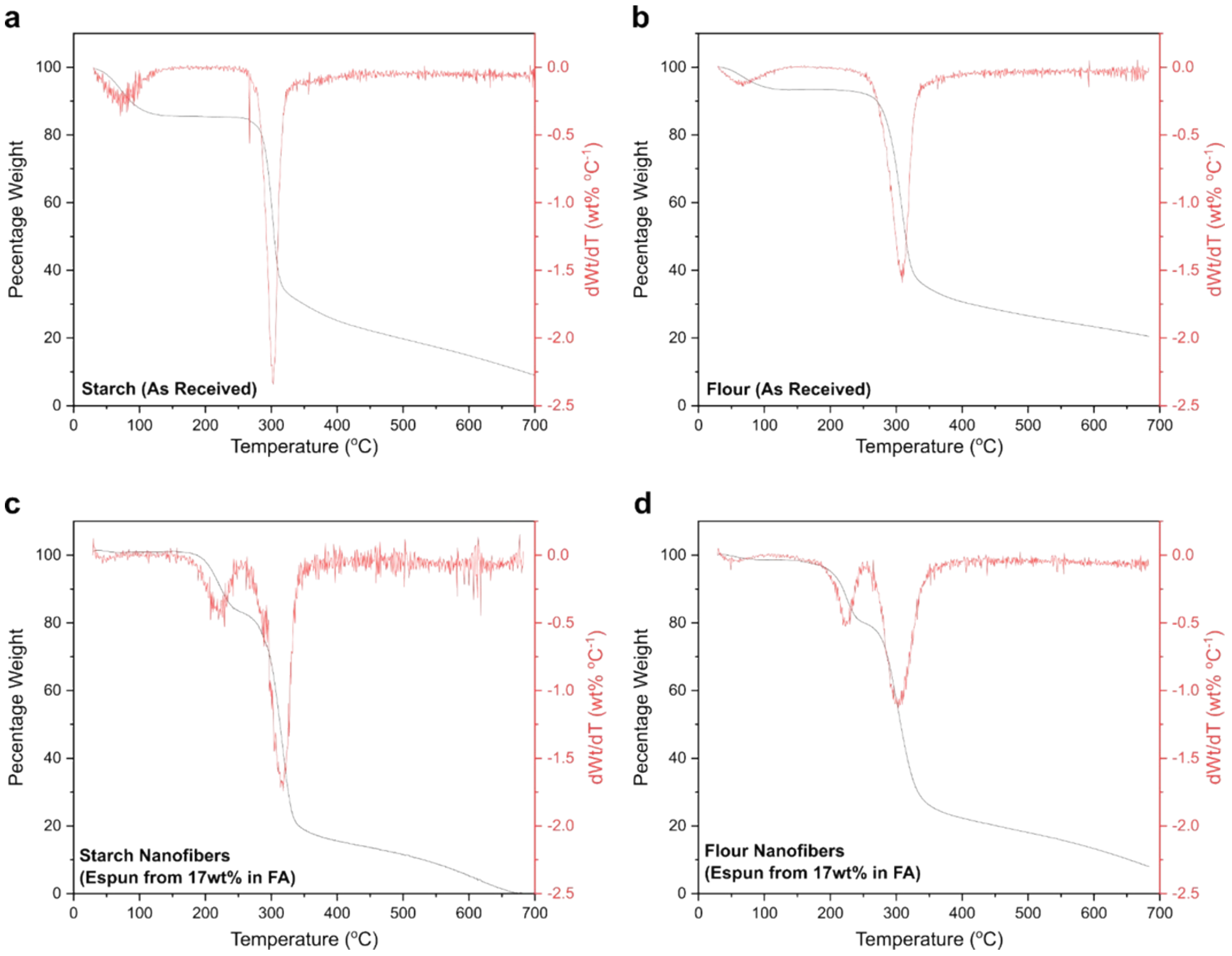

Figure S2. TGA thermograms (black) with derivatives (red) for (a) as received starch, (b) as received flour, (c) electrospun starch nanofibers, (d) electrospun flour nanofibers.

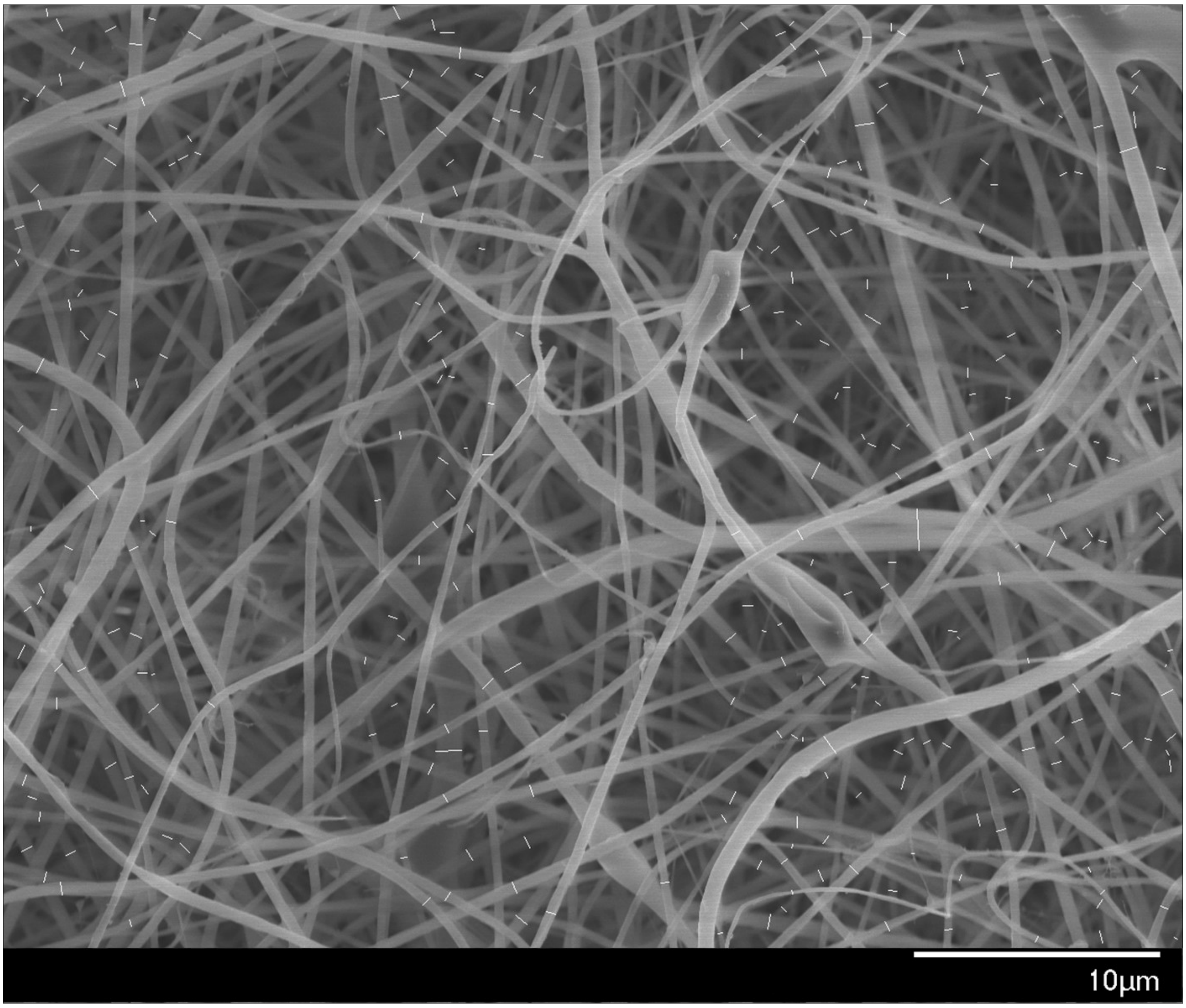

Figure S3. SEM Micrograph of flour electrospun from a 17wt% flour, with lines denoting measured fiber diameters distribution as presented in Main Text Fig 2f.

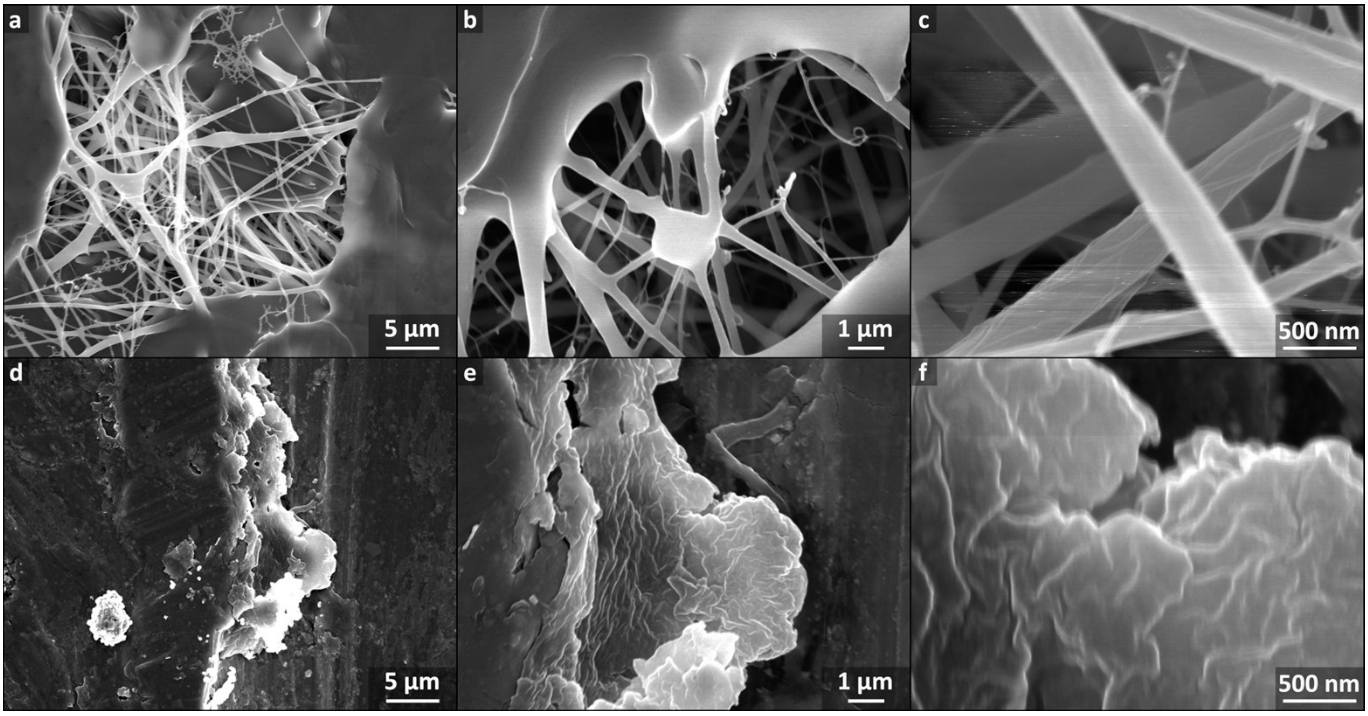

Figure S4. SEM Micrographs of flour electrospun from a 13 wt% solution in FA, stirred at room temperature for 6 hours before electrospinning.

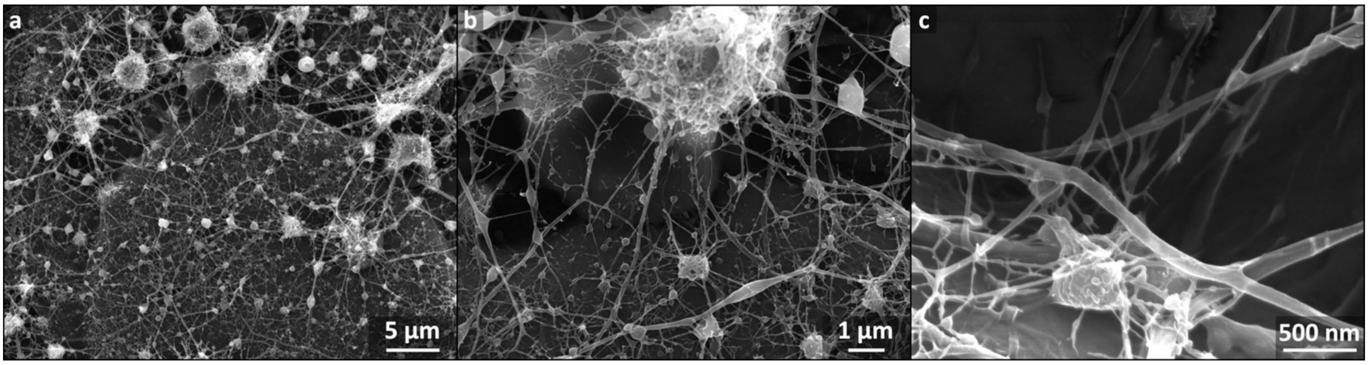

Figure S5. SEM Micrographs of flour electrospun from an 18 wt% solution in FA, stirred at 32 °C for 4 hours and cooled for 1h before electrospinning.

Supplementary Note 1 – Optimising Maize Starch Nanofibers

Dopes of starch in formic acid (FA) were dissolved with weight percentages ranging from 10 – 20 wt% in 1% increments. 10 – 15 wt% and 19 – 20 wt% starch samples were deemed insufficiently and excessively viscous, respectively, by visual inspection. The 16 – 18 wt% samples all followed the temporal trend with immediate swelling in FA to form a gel which persisted for 6 h before dissolving to a viscous liquid, with white precipitate beginning to form after ~12 h. While the general trend is reflective of that proposed by Lancuški et al.,[12] the formation of precipitate here is notably faster than any time period noted in the prior work which indicated electrospinnable dopes remained for over 24 hours. This difference is attributed to the higher content of tight-packed amylose content used (70%) in the previous report versus that in this work (27%). The independence on starch loading of the transition timings seen here are indicative of the precipitation being linked to changes in individual polymer chains modifying stability, rather than ensemble effects (e.g. reduction in saturation concentration).

By using an initial setup of 7 cm needle-collector distance and 0.8 mL h^{-1} (from previous successes on related materials), the applied voltage was tested incrementally. For the 17 wt% dope, < 18 kV leading to universal dripping and > 23 kV leading to electrospraying. All values between led to successful nanofiber formation, although 20 kV is used for the data under discussion here (Fig S6). Other dope concentrations had subtly different nanofiber windows, with 16 wt% successful between 17 – 19 kV and 18 wt% requiring 20 – 23 kV. Notably, the 16 wt% dope led to formation of some dripping throughout the sample in spite of most of the sample creating nanofibers (Fig S7).

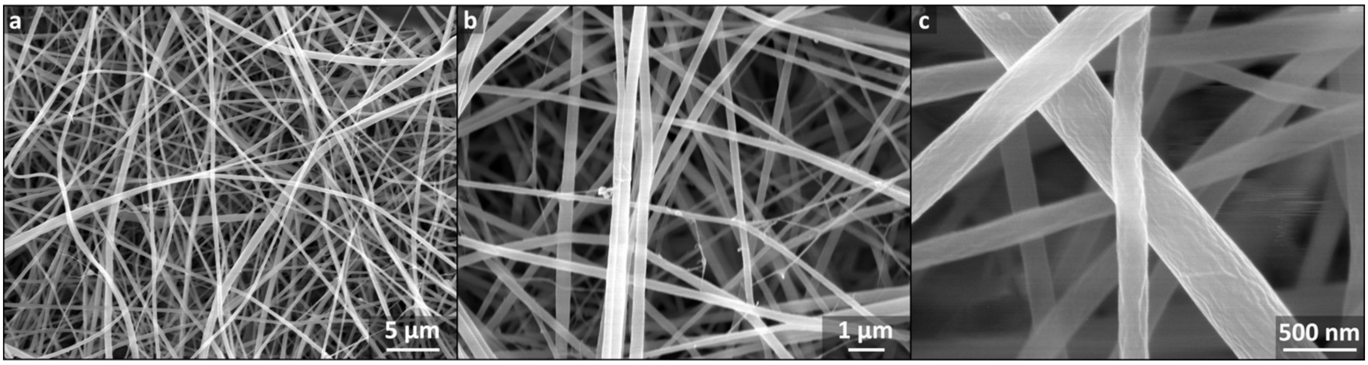

Figure S6. SEM micrographs of starch electrospun at 17 wt% in FA, stirred at room temperature for 6 hours before electrospinning.

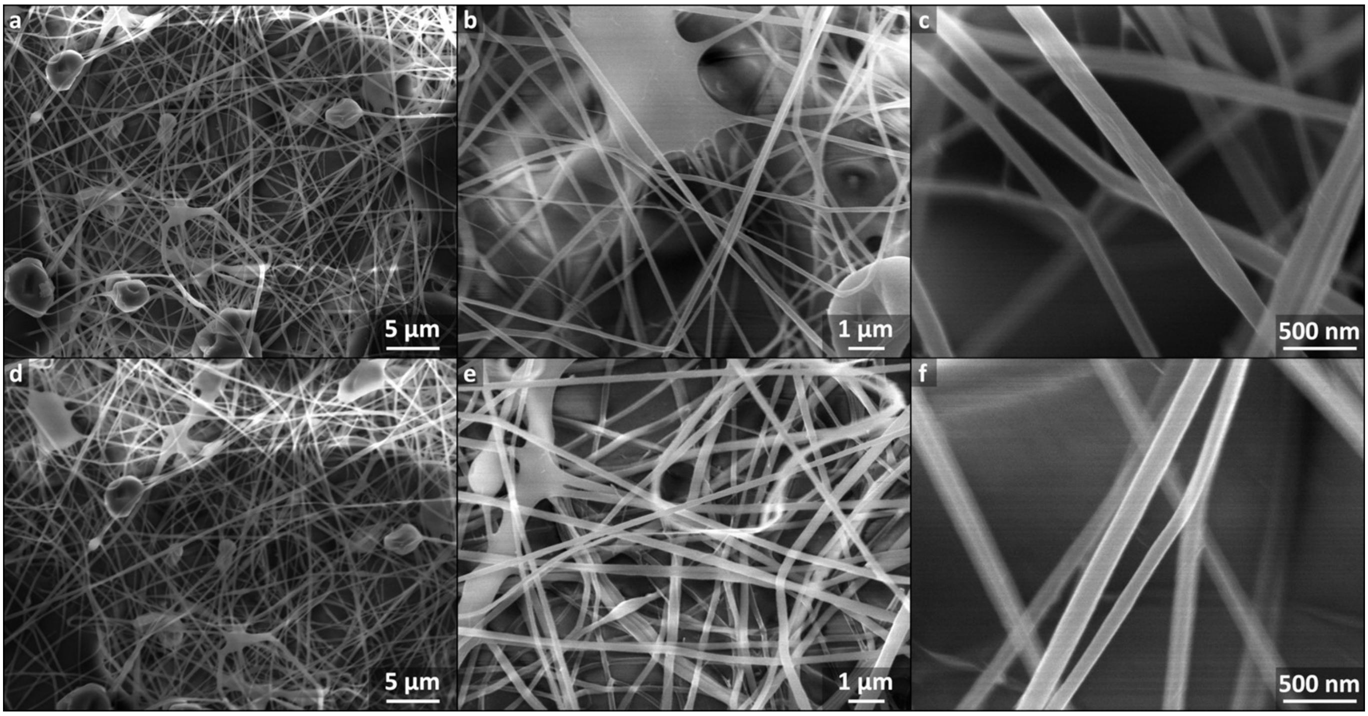

Figure S7. SEM Micrographs of starch electrospun at 16 wt% in FA, stirred at room temperature for 6 hours before electrospinning.

Supplementary Note 2 – Mechanical Testing

Mechanical tests of the as-produced films were obtained from bog-bone shaped specimens, where possible. Samples were prepared using a punch and die, ISO 527-2 Type 5B, B.083 / 084 from Zwick, UK with a gauge length of 10 mm and gauge width of 2 mm, by cutting directly into the sample on the supporting substrate which was later removed before thickness measurements and tensile testing. Thickness of the films was measured using a micrometre (131108216, RS Components) to an accuracy of ±0.001 mm. Yet, due to their production method there was significant variation in thickness from the centre (gauge region, thickest region for all samples, and used to determine the cross-sectional area of the stress indicated in table/graphs) to the ends (gripped regions) of between 16-50%, indicating that the values reported are underestimates.

Mechanical testing for 16 wt% starch and 17 wt% starch are shown in Figure S8. There is a similarity between the samples failure load which appears to be independent of the thickness and indicates that these values do not report material properties and are dependent on the samples. There is variation in stress-strain curves reported likely due to the random nature of the electrospinning process and sample thickness variation. In general, 17 wt% starch films were stiffer than 16 wt% starch films by approximately 70% this difference is likely due to improved adhesion between electrospun layers in production. A limited number of samples were prepared for 16 wt% and 17 wt% starch as the films were strongly adhered to the substrate. Thick uniform films were produced when the starch content was altered 18 wt%, however these thicker samples cracked after the application of the die. All flour-based films showed crazing from drying and suitably large samples were not possible for mechanical characterisation.

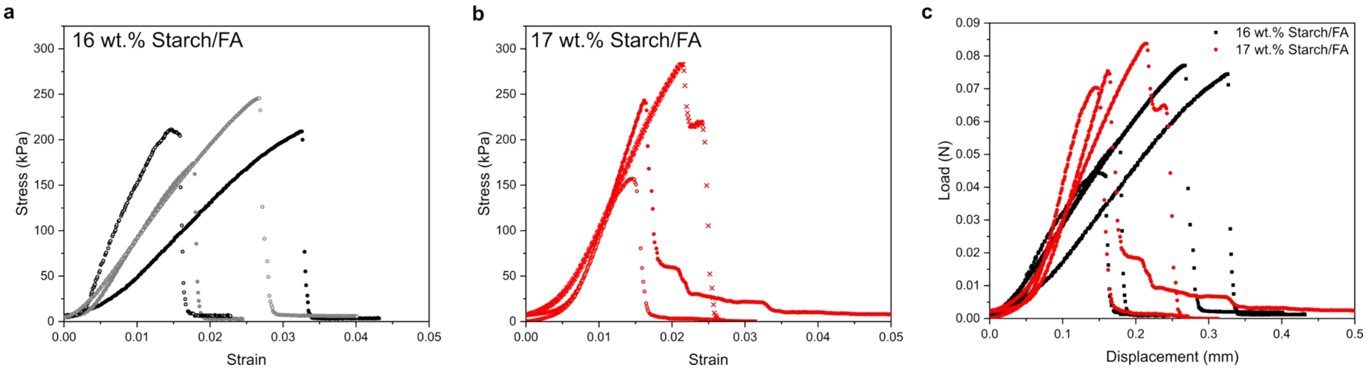

Figure S8. Tensile tests of (a) 16 wt% starch/FA and (b) 17 wt% starch/FA, with strengths defined by thickest sample region. (c) Raw data of tests highlighting common loading values at failure indicative of sample properties and not material properties.

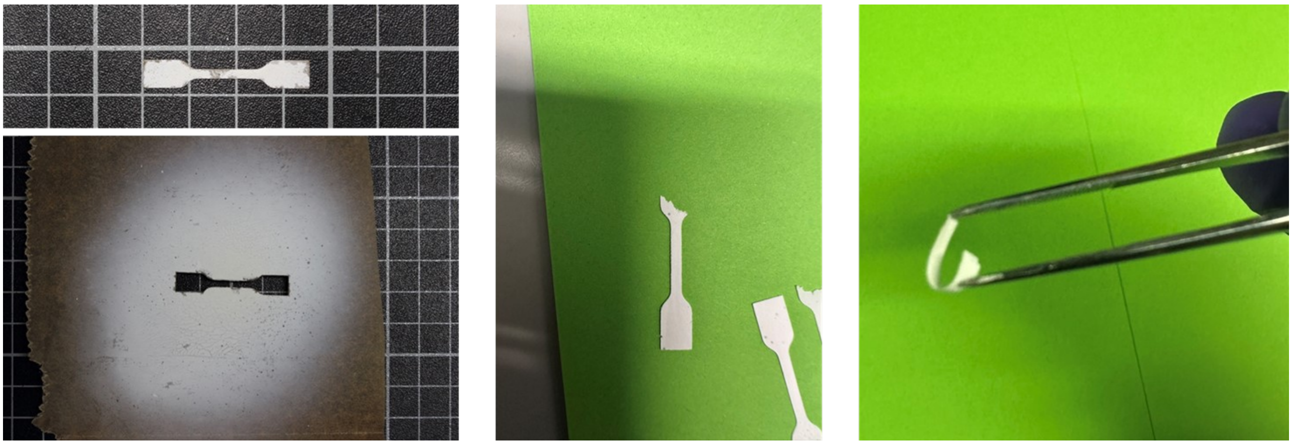

Figure S9. Digital photographs of tensile samples punched out of 16wt% starch/FA films. Samples often ruptured in grips, not in test gauge length due to non-uniformity within samples. Akin to flour samples (Main text, Fig 1cii-iii) starch sample were capable of bending.
